# A nonrandom subset of olfactory genes is associated with host preference in the fruit fly *Drosophila orena*

**DOI:** 10.1002/evl3.7

**Published:** 2017-05-09

**Authors:** Aaron A. Comeault, Antonio Serrato‐Capuchina, David A. Turissini, Patrick J. McLaughlin, Jean R. David, Daniel R. Matute

**Affiliations:** ^1^ Department of Biology University of North Carolina Chapel Hill North Carolina 27599; ^2^ Department of Biology Drexel University Philadelphia Pennsylvania 19104; ^3^ Bioko Biodiversity Protection Program Bioko Island Equatorial Guinea; ^4^ Laboratoire Evolution, Genomes, Speciation (LEGS) CNRS Gif sur Yvette Cedex France; ^5^ Université Paris‐Sud Orsay Cedex France; ^6^ Département Systématique et Evolution Museum National d'Histoire Naturelle (MNHN) UMR 7205 (OSEB) Paris France

**Keywords:** Adaptation, behavioral genetics, chemosensory, host preference, olfaction, speciation

## Abstract

Specialization onto different host plants has been hypothesized to be a major driver of diversification in insects, and traits controlling olfaction have been shown to play a fundamental role in host preferences. A diverse set of olfactory genes control olfactory traits in insects, and it remains unclear whether specialization onto different hosts is likely to involve a nonrandom subset of these genes. Here, we test the role of olfactory genes in a novel case of specialization in *Drosophila orena*. We report the first population‐level sample of *D. orena* on the West African island of Bioko, since its initial collection in Cameroon in 1975, and use field experiments and behavioral assays to show that *D. orena* has evolved a strong preference for waterberry (*Syzygium staudtii*). We then show that a nonrandom subset of genes controlling olfaction‐–those controlling odorant‐binding and chemosensory proteins–‐have an enriched signature of positive selection relative to the rest of the *D. orena* genome. By comparing signatures of positive selection on olfactory genes between *D. orena* and its sister species, *D. erecta* we show that odorant‐binding and chemosensory have evidence of positive selection in both species; however, overlap in the specific genes with evidence of selection in these two classes is not greater than expected by chance. Finally, we use quantitative complementation tests to confirm a role for seven olfactory loci in *D. orena*’s preference for waterberry fruit. Together, our results suggest that *D. orena* and *D. erecta* have specialized onto different host plants through convergent evolution at the level of olfactory gene family, but not at specific olfactory genes.

**Impact Summary**

Ecological specialization is a widespread evolutionary outcome. In insects, for example, specialization can have important economic consequences and can result in the evolution of species that damage crops or transmit disease. A major goal in evolutionary biology is therefore to understand the genes and evolutionary processes that underlie independent bouts of specialization. Here, we describe a novel case of behavioral specialization in the fruit fly *Drosophila orena*. We show that *D. orena* has evolved a strong preference for the host plant *Syzygium staudtii* and that genes acting at the periphery of the olfactory system (those encoding odorant‐binding and chemosensory proteins) show an enriched signature of positive selection and are associated with this preference. We also find a shared signature of positive selection acting on genes encoding odorant‐binding and chemosensory protein in *D. orena's* sister species, *D. erecta*, a species specialized to use fruits of *Pandanus* trees. Our study highlights how species can employ a diverse, but non‐random, subset of genes involved in olfaction during specialization into different environments.

## Introduction

Local adaptation is a fundamental evolutionary process that can drive phenotypic and genetic diversification and ultimately can be responsible for the origin of new traits and species (Darwin 1859; Schluter 2000; Rundle and Nosil 2005; Nosil 2012; Shafer and Wolf 2013). One conspicuous example of trait diversification is ecological specialization, the process whereby related species evolve to utilize different subsets of the total niche space available to them. Many groups of insects that rely on plants for food or breeding sites display such a pattern and have evolved to specialize on a small fraction of the total plant species available to them (Ehrlich and Raven 1964; Futuyma and Moreno 1988; Jaenike 1990; Nosil 2002). Given that insects are the most diverse group of animals on Earth, understanding the evolutionary processes underlying their diversification is central to our understanding of biodiversity (Jaenike 1990).

Host specialization in insects can involve the evolution of physiological, life history, and behavioral traits (Thompson 1988; Craig et al. 1989; Thompson and Pellmyr 1991; Gripenberg et al. 2010). Evolution of physiological traits can increase an organism's fitness in an environment that was previously suboptimal (e.g., *Drosophila sechellia*: Jones 2005; Dworkin and Jones 2009; Huang and Erezyilmaz 2015) and can include changes in life‐history strategies such as dispersal and reproductive potential (Southwood et al. 1974; Resetarits 1996; Denno et al. 2008). The evolution of behavioral traits can result in individuals preferentially seeking out certain environments, and in plant‐associated insects, preferences tend to be controlled by traits used to detect chemical cues generated by their preferred host plants (Tilmon 2008). Understanding how these traits evolve during host specialization is important for understanding potential trade‐offs encountered during specialization into a given ecological niche (Thompson 1988; Shoval et al. 2012; Anderson et al. 2013; Schick et al. 2015).

Chemoreception in insects is controlled by a diverse set of proteins that includes odorant‐binding proteins (OBPs), chemosensory proteins (CSPs), olfactory receptors (ORs), and gustatory receptors (GRs) (Hallem et al. 2006; Sánchez‐Gracia et al. 2009). OBPs and CSPs are small soluble proteins expressed in the sensory organs of insects that bind to distinct hydrophobic odorants and pheromones, facilitate their transport to ORs or GRs, and initiate signal transduction in sensory neurons (Vogt et al. 1991; Xu et al. 2005; Hallem et al. 2006; Sánchez‐Gracia et al. 2009). Here, we collectively refer to OBPs, CSPs, ORs, and GRs as “olfactory proteins” and their underlying genes as “olfactory genes.”

Olfactory genes or the sensory neurons in which they are expressed have been implicated in a number of cases of host specialization in insects (e.g., fruit flies: Matsuo et al. 2007; McBride 2007; Linz et al. 2013; Ramasamy et al. 2016; mosquitoes: McBride et al. 2014; aphids: Smadja et al. 2012; Duvaux et al. 2015; Eyres et al. 2016; apple maggot flies: Tait et al. 2016). Genetic variation at individual olfactory genes has also been shown to affect olfaction (Xu et al. 2005; Matsuo et al. 2007; Macharia et al. 2016), and olfactory genes are commonly thought to evolve through positive natural selection (McBride and Arguello 2007; Whiteman and Pierce 2008; Sánchez‐Gracia et al. 2009; Lavagnino et al. 2012). These findings suggest that specialization into different host environments likely involve evolution at olfactory genes. However, whether certain classes of olfactory genes may be more evolutionary labile than others, and therefore more likely to underlie different bouts of specialization, remains unknown.

A more general, and outstanding question in evolutionary biology is where phenotypic convergence occurs in terms of the hierarchy of genetic control. For example, phenotypic convergence can occur due to the same mutations, different mutations in the same gene, or different genes found in a common gene network (Manceau et al. 2010; Rosenblum et al. 2014). Identifying the genetic basis of convergence, and where it lies in the hierarchy of genetic control is important because it can shed light on constraints or biases in evolution. For example, repeated bouts of adaptation that involve the same gene (e.g., *melanocortin‐1 receptor* [*Mc1r*] in lizards [Rosenblum et al. 2010], birds [Uy et al. 2016], and mice [Steiner et al. 2007]) suggest that evolution is either constrained to use that locus, or the mutational spectrum at that locus is biased to generate adaptive phenotypes. At the other extreme, traits that are controlled by many genes may be less constrained to evolve using the same gene, especially in scenarios where there is redundancy in gene function (Yeaman 2015).

The *Drosophila melanogaster* species subgroup is well suited to study the ecological and genetic basis of host specialization. This group contains nine described species that have evolved over the last 10–15 million years to exploit a wide diversity of ecological niches (Lachaise et al. 1988; Tamura et al. 2004; David et al. 2007). Five of the species within this subgroup (*D. melanogaster*, *D. simulans*, *D. mauritiana*, *D. yakuba*, and *D. teissieri*) are considered dietary generalists, while three species (*D. sechellia*, *D. santomea*, and *D. erecta*) are thought to be dietary specialists. Of the specialist species, *Drosophila sechellia* specializes on the toxic fruits of *Morinda citrifolia* (Rubiaceae) (R'Kha et al. 1991), *D. santomea* is found in association with *Ficus chlamydocarpa fernandesiana* (Lachaise et al. 1988; Cariou et al. 2001), and *D. erecta* is seasonally abundant in regions with fruiting *Pandanus* spp. (Pandanaceae) (Rio et al. 1983; David et al. 2007). The ninth species from the clade, *D. orena*, was originally collected in 1975 in the highlands (2100 m) of the High Valley of Bafut N'Guemba, Cameroon (Tsacas and David 1978); however, the biology of this species has remained mostly unknown due to an inability to identify extant populations (Cariou 1987; David et al. 2007).

Only *D. sechellia* and *D. erecta* have been studied with respect to the genetic or physiological basis of behavioral preferences for their respective hosts. Matsuo et al. (2007) showed that genetic variation at *Obp*s affect *D. sechellia*’s preference for volatile chemicals produced by *M. citrifolia* and McBride and Arguello (2007) showed that both *D. sechellia* and *D. erecta* are losing *Or* and *Gr* genes more rapidly than related generalist species. Physiological work has also shown that *D. erecta*’s olfactory sensory neurons show increased excitation to volatiles produced by *Pandanus* fruits (Linz et al. 2013).

Here, we study the phenotypic and genetic basis of host specialization in the least known species of the *melanogaster* subgroup: *D. orena*. We use both laboratory and field experiments to show that a population of *D. orena* on the island of Bioko, West Africa displays a behavioral preference for *Syzygium staudtii* (waterberry) over other potential hosts. We then use both comparative genomic and classical genetic approaches to test the role of olfactory genes in *D. orena*’s preference for waterberry. Our results suggest that *D. orena* has evolved a strong preference for waterberry through positive selection on *Obp*s and *Csp*s. Signatures of positive evolution at olfactory loci in *D. orena*’s sister species, *D. erecta*, mirror the enrichment we observe in *D. orena* and suggest that host specialization in these two species is predisposed to involve an evolutionarily labile subset of olfactory genes that operate at the periphery of the olfactory system (*Obp*s and *Csp*s). Interestingly, the number of *Obp*s and *Csp*s that share a signature of positive selection in both *D. orena* and *D. erecta* is not greater than random expectations, indicating that convergence may occur at the level of gene family, but is not constrained to use the same locus or loci.

## Methods

### SAMPLE SITES ON THE ISLAND OF BIOKO, WEST AFRICA

We sampled *Drosophila* at five locations on the island of Bioko (Table S1). We set up five trapping stations, each consisting of one trap baited with lightly yeasted waterberry fruits, one with banana, and one with mango, at each location. We hung each trap from tree branches at an approximate height of 1 m, and collected all species present in the trap after 48 hours using an aspirator. We anesthetized each individual with triethylamine (FlyNap, Carolina Biological Supply Company) and identified and counted individuals from each species of the *melanogaster* subgroup under a light microscope. Species other than *D. orena* were immediately preserved in 70% ethanol and *D. orena* were placed in vials containing cornmeal in groups of 15–20 until later experiments.

### PREFERENCE FOR WATERBERRY AS A HOST

#### In situ estimates of host preference

We assessed host preference in *D. orena* and four other species of the *melanogaster* subgroup by testing whether each species displayed variation in the host fruit it was collected from across our five sampling stations on Bioko (Pearson's χ^2^ tests, *chisq.test* function in R).

We also tested host preference using an “eclosion” experiment, which identified the species of flies that eclosed from waterberry, *Parinari*, and fig fruits. These were the three most abundant fruits on the forest floor in regions where we collected *D. orena* on the east side of Mount Biao (1100–2020 m above sea level). We collected and placed fruit in 259 mL glass bottles supplemented with a pupation substrate (Kimwipes, Kimberly Clark, Roswell, GA), for a total of 15 bottles of each type of fruit. Larvae and pupae were allowed to develop within the bottles and upon emergence we identified and counted adults belonging to the *melanogaster* subgroup. We restricted our counts to male flies because species‐specific male traits are easy to unambiguously identify relative to female traits in living individuals in the field (Markow and O'Grady 2006; Orgogozo and Stern 2009). We tested whether eclosion rates for each species varied across the three substrates with Pearson's χ^2^ tests.

#### Lab‐measured preference for waterberry fruits

We next tested the host preference of *D. orena*, *D. melanogaster*, *D. simulans*, *D. teissieri*, and *D. yakuba* using food‐choice behavioral assays. We collected adult flies from the trapping stations described above and maintained them in species and sex‐specific vials containing cornmeal food for 4 days prior to behavioral assays to allow them to recover from the anesthesia. In total, we assayed 210 *D. orena*, 512 *D. melanogaster*, 410 *D. simulans*, 422 *D. teissieri*, and 398 *D. yakuba*. Prior to behavioral assays, we placed an average of 26 flies of the same species and sex (range: 21–31) into empty vials with a source of water (i.e., hydrated cellulose acetate plugs, Genesee Scientific) and starved them for 12 hours overnight. The following morning, we connected the vials containing flies to vials containing cornmeal fly food on one side and waterberry fruit on the other (see Turissini et al. 2017 for additional details). We let flies choose a side for three hours and scored the number of flies in each vial (either waterberry, cornmeal, or center) at the end of the assay. The proportion of flies in the waterberry vial relative to the cornmeal vial was used as an estimate of food preference.

We tested whether *D. orena* preferred waterberry more than the other four species by fitting a generalized linear model (GLM; *glm* function in the R library “stats”) with binomially distributed error that modeled the proportion of individuals in the waterberry vial as a function of species, sex, and the interaction between species and sex. We also analyzed the ratio of individuals choosing waterberry over those choosing cornmeal, for each species, using exact binomial tests (EBTs; *binom.test* function in R) with the null expectation that a fly was equally likely to choose waterberry or cornmeal. These two tests allowed us to ask whether *D. orena* has a stronger preference for waterberry compared to other closely related species and if they have a general preference for waterberry over cornmeal, respectively.

### PERFORMANCE ON WATERBERRY ACROSS THE *MELANOGASTER* SUBGROUP

In addition to preference, we estimated performance of *D. orena*, *D. yakuba*, *D. teissieri*, *D. melanogaster*, and *D. simulans* when raised in each of six different host environments: mango, fig, banana, cornmeal, waterberry, and instant *Drosophila* medium (Carolina Biological, Burlington, NC). Females (*N* = 600 of each species) were individually mated to conspecific males and pooled into groups of 10 individuals (*N* = 60 groups per species). We randomly assigned 10 groups of each species to each of the six host environments. After 10 days of laying eggs we removed the females from the vials and added a Kimwipe (Kimberly Clark) dampened with 0.5% propionic acid (to prevent fungal growth) as a pupation substrate. We counted the number of adult flies that eclosed from each replicate vial over the following 3 weeks (i.e., until no more flies emerged) as a measure of performance. This approach integrates performance across the propensity of females to lay eggs on a given substrate and the ability of those eggs to hatch, larvae to develop and pupate, and pupae to successfully eclose into adult flies.

To determine whether performance varied across host environments we first fitted a GLM testing for an interaction between species and host environment. We assessed significance of this interaction with a likelihood ratio test that compared the fit of the full model (fixed effects: species, host environment, and the interaction between species and host environment) to one lacking the interaction term. This analysis showed a highly significant interaction between species and host environment (see Results) on composite performance; therefore, we also looked at variation in performance across host environments for each species independently by fitting a GLM where the number of eclosing adults was the response and host environment was the fixed effect. We then compared performance between environments using Tukey's post‐hoc contrasts. Because we were specifically interested in performance when raised on waterberry fruits, we only report pairwise contrasts between waterberry and each of the other host environments. GLMs were fitted assuming Poisson‐distributed error.

### THE ROLE OF OLFACTORY GENES IN HOST SPECIALIZATION

Because our phenotypic results indicate that *D. orena* has evolved a strong preference for waterberry fruits (see Results), we used both comparative genomic and classical genetic approaches to determine the role of genes associated with olfaction in this behavioral specialization.

#### Evolutionary rates of olfactory genes

We resequenced the genome of a single female *D. orena* and mapped the reads to the *D. erecta* reference genome to a mean per‐site coverage of 26.16X (*D. erecta* is *D. orena*’s sister species; see SI for details). We computed the ratio of synonymous (*K_s_*) to nonsynonymous (*K_a_*) substitutions (*ω*) in both the *D. orena* and *D. erecta* lineages using codeml from the PAML 4.8 package (Yang 2007). We computed *ω* for 13,605 *D. erecta* genes using a three‐species alignment of *D. orena*, *D. erecta*, and *D. yakuba* and polarized substitutions as being derived in either the *D. orena* or *D. erecta* lineage, whenever possible, using *D. yakuba* as the outgroup (*D. yakuba* is the sister species of *D. orena* and *D. erecta*; see SI for information regarding the *D. yakuba* sequence we used in our analysis). We categorized each gene as being subject to positive selection if *ω* was greater than or equal to 1 (Li et al. 1985; Yang and Nielsen 2000). Sites where both *D. orena* and *D. erecta* were derived (i.e., different alleles for *D. orena*, *D. erecta*, and *D. yakuba*) were not used in the lineage‐specific ratios because we could not infer the ancestral state.

Of the 13,605 genes, 12,387 have a known homolog in *D. melanogaster*. We used our estimate of *ω* for these 12,387 genes to test whether genes annotated as *Obp*s (46 of the 12,387 genes), *Csp*s (18 genes), *Gr*s (59 genes), or *Or*s (60 genes) (Graham and Davies 2002; Hekmat‐Scafe et al. 2002; Vieira et al. 2007) showed elevated rates of adaptive evolution across the *D. orena* and *D. erecta* dyad or along the *D. orena* or *D. erecta* branches. We tested whether the fraction of a given class of olfactory genes with *ω* ≥ 1 was greater than the genomic background with Fisher's exact tests (FETs; *fisher.test* function in R). We also compared the distribution of *ω* for each of the classes of olfactory genes to that of the remaining genes in our dataset using Wilcoxon rank sum tests (WRSTs; *wilcox.test* function in R).

Because we have estimates of *ω* for both *D. orena* and *D. erecta*, we tested whether any olfactory genes showed evidence of positive selection along both these lineages. For each class of olfactory gene, we determined whether the proportion of genes with *ω* ≥ 1 along both the *D. orena* and *D. erecta* lineage was enriched relative to random expectations. To test for convergent positive selection, we only considered genes with *ω* ≥ 1 as a result of different substitutions along the *D. orena* and *D. erecta* branches. We took this approach because we cannot rule out the possibility that a shared substitution evolved independently along both the *D. orena* and *D. erecta* branches or once along the branch leading from the common ancestor of the clade ((*D. orena*, *D. erecta*), *D. yakuba*) to the split between *D. orena* and *D. erecta*. We used randomization tests to assess whether the overlap in genes with *ω* ≥ 1 was greater than expected by chance. These tests compute the expected number of “convergent” genes of a given class relative to the observed number of genes in that class with evidence of positive selection along the *D. orena* and *D. erecta* branches. We generated 10,000 randomized data sets for each class of gene and calculated empirical *P*‐values as the proportion of the randomized samples where the number of convergent loci (i.e., loci with *ω* ≥ 1 in both lineages) was equal to or greater than the observed number between *D. orena* and *D. erecta*. Finally, we tested for a correlation (Spearman's) between estimates of *ω* along the *D. orena* and *D. erecta* branches for each class of olfactory gene (*cor.test* function in R).

In addition to our primary analysis of *D. orena* and *D. erecta*, we calculated *ω* for two additional species of the *melanogaster* subgroup: *D. santomea* and *D. yakuba* (see SI for details). This analysis allowed us to ask whether patterns of enrichment in *ω* we observe for olfactory genes in *D. orena* and *D. erecta* are shared in other specialist (*D. santomea*) or generalist (*D. yakuba*) species. If patterns of *ω* were shared for a given group of olfactory genes across all species, this would indicate that evolutionary rates we observe in *D. orena* are not related to host specialization, but are instead due to some intrinsic property of the gene family itself (e.g., if olfactory‐binding proteins evolve rapidly in all lineages). We tested whether the proportion of olfactory genes of a given family with *ω* ≥ 1 was enriched relative to genome‐wide expectations (i.e., compared to “nonolfactory” genes) for both *D. santomea* and *D. yakuba* using FETs.

#### Complementation tests

We took advantage of the fact that *D. melanogaster* females can be forced to hybridize with *D. orena* males to conduct complementation tests that isolate the effect of *D. orena* alleles on the behavioral preference for waterberry. (The rate of hybridization is ∼1/400 trials.) We focused on *Obp*s because our analysis of *ω* identified the largest number of candidate loci for this class (Results). Consistent with previous food‐choice experiments in hybrid *Drosophila* (Turissini et al. 2017), *D. melanogaster* × *D. orena* F1 hybrid females are, in general, less able to locate food than their parents (FETs: F1s compared to *D. melanogaster*: odds ratio = 14.99, *P* < 1.0 × 10^−11^; F1s compared to *D. orena*: odds ratio = 7.53; *P* < 1.0 × 10^−7^). More importantly, the F1 hybrids that do locate food prefer cornmeal to waterberry (EBT: 26/30 chose cornmeal; *P* < 1.0 × 10^−4^; Fig. 4B) and do not differ from *D. melanogaster* in their preference (FET: odds ratio = 0.36; *P* = 0.0609). *Drosophila orena* preference alleles are, therefore, mostly recessive to *D. melanogaster* alleles, allowing us to test their effect in hemizygous hybrids that carry a deficiency along the chromosome inherited from *D. melanogaster*.

We used seven *D. melanogaster* deficiency (*df*) stocks (i.e., lines containing chromosomal aberrations resulting in a deleted stretch of their genome) that spanned nine *Obp* loci with *ω* > 2 along the *D. orena* branch (Table S2) and have breakpoints that have been molecularly characterized. Five of these *df*s span a single *Obp* locus and two span three *Obp*s. Both *df*s spanning more than one *Obp* covered three loci (*Obp56e*, *Obp56g*, and *Obp56i*; and *Obp57a*, *Obp57b*, and *Obp57e*); therefore, we can only assess the effect of these alleles jointly. The effect of *Obp19b*, *Obp22a*, and *Obp83cd* were assessed using three unique *df*s and those of *Obp22a* and *Obp83cd* were each assessed using two independent but overlapping *df*s. We also attempted to cross deficiency stocks spanning five additional *Obp*s (*Obp50a*, *Obp991*, *Obp99c*, *Obp99d*, and *Obp93a*), but obtained no hybrid progeny. Each *Obp df* was paired with a *df* that spanned a region adjacent to the targeted *Obp*. This set of *df*s acted as a genetic “control” to verify that not all *df*‐carrying hybrids were attracted to waterberry fruit. Each *df* spanned additional loci that are not predicted to affect the olfactory system (Table S3). All *df*s were maintained over a Balancer chromosome (*Bal*; *FM7* for *X*‐linked deficiencies, *CyO* for deficiencies on the second chromosome, and *TM3*, *Sb* for deficiencies on the third chromosome). We crossed females from *D. melanogaster* stocks (*Bal/df*) to *D. orena* males. The cross between a *D. melanogaster Bal/df* female and a *D. orena* male produces *mel/ore* F1 female hybrids with two genotypes: those carrying a single copy of the *D. orena Obp* allele over a melanogaster chromosome that is deficient with respect to this allele (*df*/*ore*) and those carrying a copy of the *D. melanogaster* Balancer chromosome and the *D. orena Obp* allele (*Bal*/*ore*). The complementation test we carried out is therefore similar to quantitative deficiency mapping (Anholt and Mackay 2004). We tested whether the flies expressing the *D. orena* allele (*df/ore*) were more attracted to waterberry fruit than their sisters that carried both *D. melanogaster* and *D. orena* alleles (*Bal*/*ore*). We obtained at least 50 hybrid females for each genotype and ran them through behavioral assays that tested their propensity to choose waterberry as a resource as described in “Lab‐measured preference for waterberry fruit.” For each *df* we compared the proportion of *df/ore* hybrids that chose waterberry fruit over cornmeal to the same proportion of *Bal/ore* hybrids using FETs.

## Results

### DISCOVERY OF *D. orena* ON THE ISLAND OF BIOKO, WEST AFRICA

**Figure 1:**
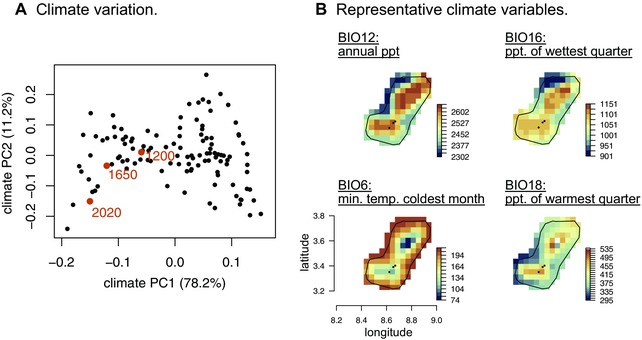
*Drosophila orena*’s climatic niche. (A) Climatic space across the island of Bioko and the position in this space for sites where we collected *D. orena* (red points; elevation in meters above sea level is shown beside these points). (B) Variation in four representative bioclimatic variables that loaded heavily on the PC axes shown in (A) across the island of Bioko (ppt. = precipitation; min. = minimum; temp. = temperature).

**Figure 2:**
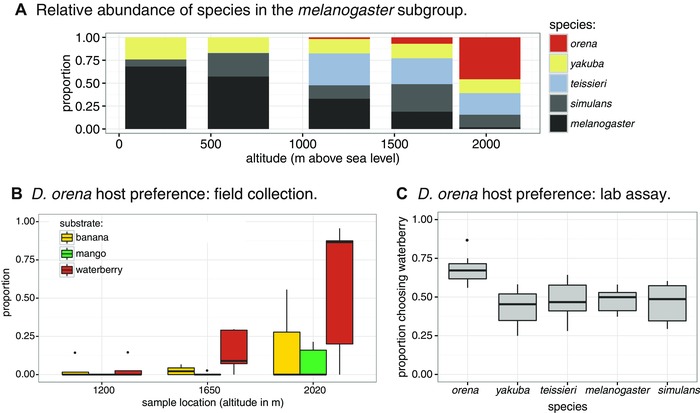
*Drosophila orena* is a high altitude specialist found on waterberry. (A) *D. orena* are only found at high elevations and are the primary species of the *melanogaster* species subgroup found at high elevations. The specific altitudes of the five sample locations are reported in Table S1. (B) At the high elevation sites, *D. orena* were preferentially collected from traps baited with waterberry. (C) Of the five species from the *melanogaster* subgroup we sampled on Bioko, *D. orena* was the only one showing a preference for waterberry fruit in food‐choice behavioral assays when given the choice between waterberry and a cornmeal substrate.

We initially sampled a single *D. orena* female in 2009 from a hanging banana trap on Bioko (DRM). J.R. David inspected this individual and concluded it was *D. orena* based on morphology. The genital morphology of male *D. orena* is distinct from other species of the *melanogaster* subgroup: they have a large truncated phallus in the shape of a boomerang, basal phallic hooks, and no epandrial posterior lobes (Yassin and Orgogozo 2013). They also differ from their sister species *D. erecta* in that the latter has a hook shaped phallus curved dorsally. Female *D. orena* can be identified based on the presence of a sclerotinized vulval shield (Yassin and Orgogozo 2013). The *D. orena* that we report and analyze here were collected during an expedition to the island of Bioko in 2013. All *D. orena* we collected were sampled above 1200 m at cool and wet sites relative to the rest of the island (Figs. 1 and 2A; SI). No *Pandanus* spp. plants, the preferred host of *D. erecta* was found in Bioko, decreasing the possibility of our collections being *D. erecta*. We confirmed that the flies we sampled were not *D. erecta* by performing controlled crosses between the isofemale lines we established and the one *D. orena* line that was collected from Cameroon in 1975. All flies mated readily and produced fertile offspring (data not shown).

### PREFERENCE FOR WATERBERRY AS A HOST

We first tested *D. orena*’s host preference in the field. Across the three locations where we collected *D. orena* (Figs. 1A, 2A and 2B), over 80% were collected over waterberry, while we tended to collect all other species of the *melanogaster* subgroup banana or mango (Table 1). This indicates that *D. orena* has a strong preference for waterberry. The number of male flies emerging from waterberry, *Parinari*, and fig fruit support this conclusion: *Drosophila orena* was the only species where the majority of individuals emerged from waterberry fruits (Table 2). At least one *D. orena* emerged from 9 of the 15 waterberry replicates and two of the 15 fig replicates, while none emerged from *Parinari*. Despite this strong bias to emerge from waterberry, it is possible that *D. orena* utilizes species of hosts we did not sample, and/or vary in their resource use throughout the year, as has been observed in *D. erecta* (Rio et al. 1983; David et al. 2007). While at least one individual of each of the other *melanogaster* subgroup species emerged from waterberry, the majority of individuals of *D. yakuba*, *D. melanogaster*, and *D. simulans* emerged from figs, while *D. teissieri* emerge mainly from *Parinari* fruits (Table 2).

**Table 1:** *Drosophila orena* strongly prefer waterberry fruit over other suitable substrates

Species	Waterberry	Banana	Mango	χ^2^	df	*P*‐value
*D. melanogaster*	618	614	643	0.39	2	0.823
*D. simulans*	314	334	290	1.56	2	0.468
*D. teissieri*	70	513	197	200.88	2	0.000
*D. yakuba*	186	299	306	18.78	2	8.0 × 10^−5^
*D. orena*	154	27	12	87.96	2	0.000

The number of flies caught over each type of fruit was pooled across sample locations and replicates (see main text for details; Table S4 for numbers grouped by the elevation collected from).

**Table 2:** Species from the *melanogaster* subgroup vary in their frequency of host use

Species	Waterberries	*Parinari*	Figs.	χ^2^	*P*‐value
*D. melanogaster*	5	12	39	34.5	3.17 × 10^−8^
*D. simulans*	4	10	16	7.2	2.73 × 10^−2^
*D. teissieri*	2	45	3	39.9	2.02 × 10^−16^
*D. yakuba*	10	1	22	20.2	4.15 × 10^−5^
*D. orena*	15	0	2	23.4	8.25 × 10^−6^

Species and number of individuals that eclosed from waterberries, *Parinari*, and figs that we collected on the island of Bioko.

Finally, in lab‐based choice assays, *D. orena* was more likely to move toward waterberry than any of the four other species (GLM: species term; deviance = 35.83; *P* = 3.13 × 10^−7^; Tukey contrast of *D. orena* versus each other species: all *P* < 0.05, except for with *D. melanogaster*, where *P* = 0.051; Fig. 2C). *Drosophila orena* was also the only species to show a general preference for waterberry fruit over cornmeal (EBT: flies attracted to waterberry fruit/total: *D. orena* = 144/210; *P* < 1 × 10^−15^; *D. melanogaster* = 250/512, *P* = 0.6269; *D. simulans* = 187/410; *P* = 0.08377; *D. yakuba* = 180/398, *P* = 0.06351; *D. teissieri* = 200/422; *P* = 0.3067; Fig. 2C). Together, sampling in the field, rates of emergence from field‐collected hosts, and lab‐based behavioral assays all indicate that *D. orena* has a strong preference for waterberry.

### PRODUCTION OF OFFSPRING ON WATERBERRY ACROSS THE *MELANOGASTER* SUBGROUP

**Figure 3:**
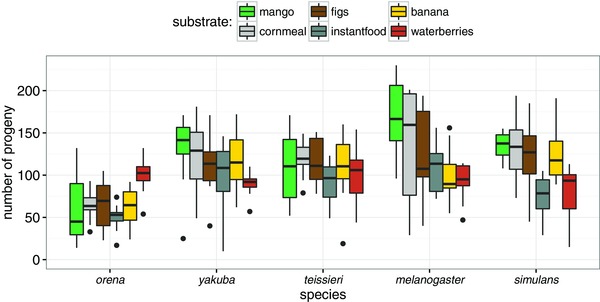
Performance on different dietary substrates. Of the five species in the *melanogaster* subgroup we sampled on Bioko, *D. orena* was the only species showing higher levels of performance on waterberry when compared to five different host environments (i.e., substrates).

Specialist species of *Drosophila* commonly utilize a host that is either toxic or nutrient poor compared to other potential hosts (e.g., (Heed and Kircher 1965; Etges 1993; Jones 2005; Dworkin and Jones 2009; Linz et al. 2013). We assessed performance on waterberry for five species of the *melanogaster* subgroup. The interaction between host environment and species has a large effect on performance (LRT: χ^2^ = 557.31, *P* < 10^−15^; Fig. 3). When contrasting the production of offspring in different environments for each species separately, we find that *D. orena* is the only species that performs best when raised on waterberry (Table 3). *Drosophila melanogaster*, *D. simulans*, *D. teissieri*, and *D. yakuba* typically performed worse on waterberry than when raised on mango, cornmeal, fig, instant food, or banana (Table 3), indicating that waterberry was a suboptimal host for all these species.

**Table 3:** Tukey's pairwise comparisons of composite performance when raised on waterberries versus five different food substrates

Comparison	*D. orena*	*D. yakuba*	*D. teiss*.	*D. melan*.	*D. simu*.
Waterberry vs. mango	**9.885**	−*8.8*	−1.911	−*14.682*	−11.79
Waterberry vs. cornmeal	**8.885**	−*7.388*	−*4.355*	−*9.219*	−*11.202*
Waterberry vs. fig	**8.271**	−*4.949*	−*3.7*	−*6.85*	−*10.015*
Waterberry vs. instant food	**12.463**	−0.07146	1.809	−*3.333*	1.222
Waterberry vs. banana	**9.127**	−*0.27412*	−2.061	−2.067	−*9.88*

**bold** text: waterberry > other at *P* < 0.05; *italic* text: waterberry < other at *P* < 0.01; plain text = no significant difference.

Values represent test statistics (Z statistics) for each substrate comparison for each of the five species we focus on in this manuscript.

### THE ROLE OF OLFACTORY GENES IN HOST SPECIALIZATION

#### Evolutionary rates of olfactory genes

**Figure 4:**
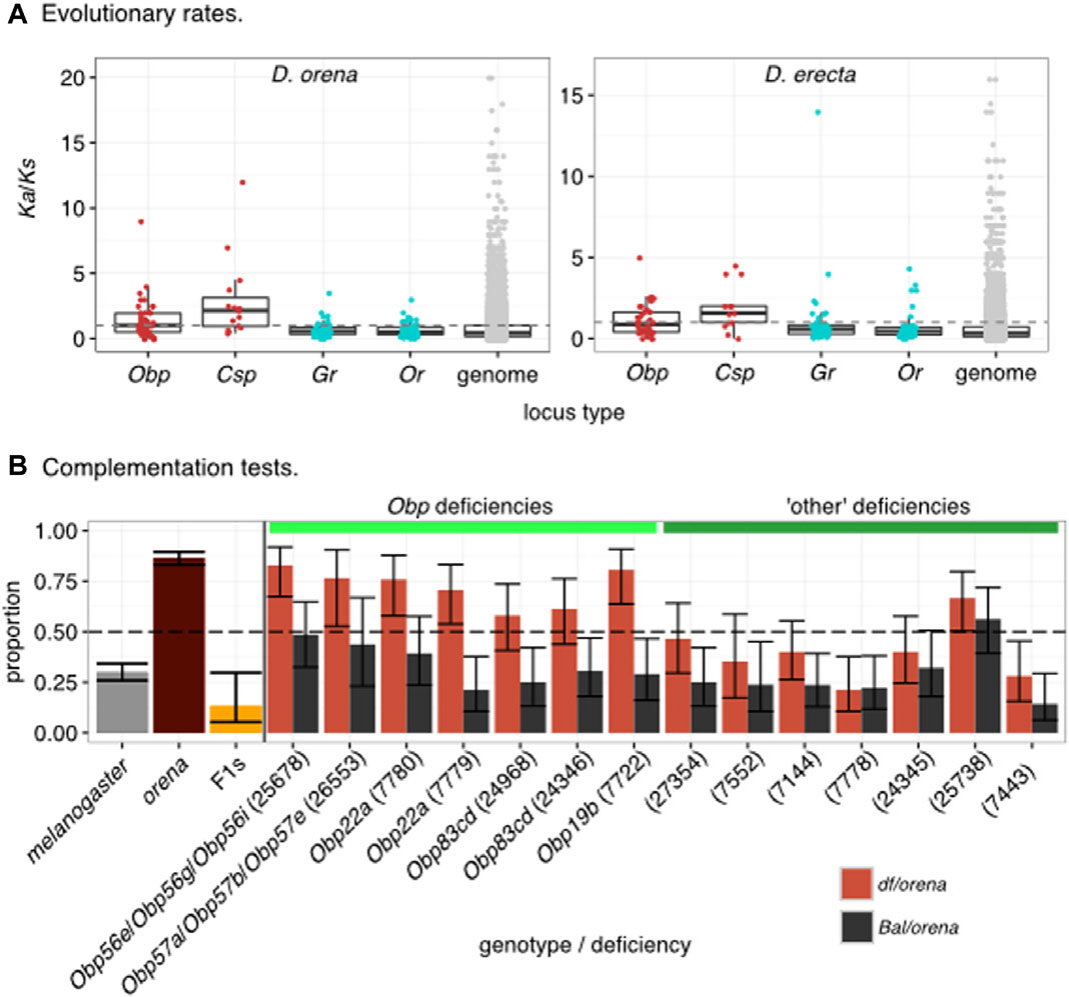
Comparative genomics and deficiency mapping show that *Obp* and *Csp* alleles underlie *D. orena*’s preference for waterberry. (A) Estimates of *Ka*/*Ks* for genes of different types (see main text for descriptions of the different “types”). *Obp*s and *Csp*s (red points) are enriched for the proportion of loci with *Ka*/*Ks* ≥ 1 compared to *Gr*s, *Or*s (light blue points), and the nonolfactory genes (“genome”; gray points) (FETs; all *P* < 0.05). The overall distributions of *Ka*/*Ks* values for *Obp*s and *Csp*s are also greater than those of *Grs*, *Or*s, and nonolfactory genes (Wilcoxon rank sum tests; all *P* < 0.0001). (B) Bars show the proportion of flies of a given genotype that choose waterberry versus cornmeal in food‐choice behavioral assays. The proportion of pure species and their F1 hybrids (three leftmost bars) indicate that *D. orena* preference alleles are recessive to *D. melanogaster* alleles. When recessive *D. orena* alleles present over a *D. melanogaster* deficiency (*df/ore*), hybrids prefer waterberry over cornmeal when compared to *D. orena* alleles found over *D. melanogaster* balancer chromosomes (*Bal/ore*). This pattern was not observed for seven genetic “controls” (“other” deficiencies) located adjacent to the *Obp* deficiencies. Error bars were computed using the “binconf” function of the *Hmisc* R library and represent 95% confidence intervals. See Table S6 for data.

Given the strong preference of *D. orena* for waterberry, we hypothesized that olfactory genes would show a signature of adaptive (or at least accelerated) evolution since the split between *D. orena* and its sister species, *D. erecta*. We measured *ω* along both lineages for 46 *Obp*s, 18 *Csp*s, 59 *Gr*s, and 60 *Or*s (Table S6) and found that *Obps* and *Csp*s are more likely to show signatures of positive selection than nonolfactory genes in both *D. orena* and *D. erecta*: 26 (56.5%) and 21 (45.6%) *Obp*s and 11 and 10 *Csp*s have *ω* ≥ 1 along the *D. orena* and *D. erecta* branches, respectively, compared to 2958 (24.2%) and 2102 (17.2%) of 12,204 nonolfactory genes (Fig. 4A). When comparing between classes of olfactory genes, the proportion of loci with *ω* ≥ 1 does not differ between *Obp*s and *Csp*s in either species (FETs: *D. orena*: odds ratio = 0.77; *P* = 0.64, *D. erecta*: odds ratio = 0.65; *P* = 0.45) but is greater for both *Obp*s and *Csp*s when compared to *Gr*s and *Or*s (FETs; Fig. 4A).

We next addressed the possibility of convergent positive selection within olfactory gene families. We found 12 *Obp*s (26.1%), 8 *Csp*s (44.4%), 7 *Gr*s (11.9%), and 2 *Or*s (3.3%) versus 1257 of 12,204 nonolfactory genes (10.3%) with *ω* ≥ 1 in *D. orena* and *D. erecta*. The proportion of genes of each family with evidence of positive selection in *D. orena* and *D. erecta* was only enriched for *Gr*s (randomization tests: expected median numbers [empirical *P*] for *Obp*s: 12 [0.59]; *Csp*s: 7 [0.41], *Gr*s 2 [0.0003], and *Or*s 1 [0.42]). Estimates of *ω* for *Obp*s and *Csp*s are not correlated between *D. orena* and *D. erecta* (*ρ* = 0.28 and 0.20; *P* = 0.07 and 0.50, respectively) but they are for *Gr*s and *Or*s (*ρ* = 0.62 and 0.46; *P* = 1.94 × 10^−7^ and 3.86 × 10^−4^, respectively). This result suggests that *Obps* and *Csps* have been more evolutionarily labile than *Grs* and *Ors* in *D. orena* and *D. erecta*. Our comparative analysis of *ω* shows that *Obp*s and *Csp*s display elevated rates of nonsynonymous substitution, suggesting that they have repeatedly been involved in host‐use specialization; however, the specific loci used in a bout of specialization do not tend to overlap more than expected by chance.

In the related generalist species *D. yakuba*, only *Csp*s show an enrichment in *ω* compared to the genome‐wide expectation (3/6 vs 823/5611; FET: *P* = 0.045; Table S7); however, this result is based on only 6 *Csp* loci that have one or more derived synonymous substitution along the *D. yakuba* branch. For the related specialist species *D. santomea*, *Csp*s (6/12; *P* = 0.012), *Gr*s (13/28; *P* < 0.001), and *Or*s (14/35; *P* = 0.003) all have enriched estimates of *ω* compared to genome‐wide expectations (1185/6517), but *Obp*s does not (0/10; *P* = 0.22) (Table S7). These results support the idea that specialist species have accelerated rates of evolution at olfactory genes, but the specific loci and locus family varies across independently evolved behavioral preferences.

#### Complementation tests

We tested whether *df*‐carrying hybrids (i.e., where the *Obp^orena^* allele is hemizygous: *df/ore*) are more attracted to waterberry fruit than their *Bal*‐carrying sisters (i.e., where the *orena* allele is accompanied by a *melanogaster* allele: *Bal/ore*). For six out of the seven *Obp‐*spanning *df*s we screened, this was the case (FETs; all *P* < 0.015; Fig. 4). F1 hybrids carrying the *df* that spanned the three *Obps* at band 57 (*Obp57a^ore^, Obp57b^ore^*, and *Obp57e^ore^*) (i.e., *Df(2R)BSC702/ore*) were marginally more attracted to waterberry fruit than their *Bal/ore* siblings (FET: odds ratio = 0.25; *P* = 0.080). Despite this small difference between the two chromosomal types, the *df*/*ore* F1s showed a slight preference for waterberry fruit compared to the random expectation (EBT: 13/17 individuals chose waterberry; *P* = 0.049), suggesting that alleles at one (or more) of the three loci affect the preference for waterberry fruit. For seven “control” deficiencies, none of the *df/ore* F1s showed a preference for waterberry (Fig. 4; Table S8). These results confirm that *D. orena Obp* alleles that have evolved through strong positive selection (*ω* > 2) can causally affect levels of preference for waterberry fruits, however further validation of the *Obp*s, *Csp*s, *Gr*s, and *Or*s with evidence of positive selection are required.

## Discussion

Our results show that *D. orena* found on the island of Bioko have evolved a strong preference for waterberry fruits over other suitable substrates. This finding adds to the growing body of work that describes the natural history and evolution of the nine species within the *melanogaster* species subgroup (Lee and Watanabe 1987; David et al. 2007; Dworkin and Jones 2009; Linz et al. 2013; Yassin et al. 2016). Together, these studies suggest that host specialization within the *melanogaster* subgroup is not a rare phenomenon, with four of the nine species, and one population of the generalist species *D. yakuba* (Yassin et al. 2016), primarily being found on a single host species. Specialization may not be rare in *Drosophila*: upwards of 70% of Hawaiian *Drosophila* are estimated to be specialists (Heed 1971), other *Drosophila* have specialized on rotting cacti (Morales‐Hojas and Vieira 2012) or flowers (Brncic 1983), while others have evolved anatomical specializations for hard‐bodied fruits (e.g., Atallah et al. 2014). Despite these examples, a formal test of the frequency of specialization across *Drosophila* is, to our knowledge, yet to be conducted.

Unlike some examples of host specialization in *Drosophila* (e.g., *D. sechellia* [Farine et al. 1996; Dworkin and Jones 2009] and *D. pachea* [Heed and Kircher 1965; Lang et al. 2012]), waterberry fruit is not toxic to any of the other four species of the *melanogaster* species subgroup found on Bioko (Fig. 3). However, all four species we assayed (other than *D. orena*) performed best on a substrate other than waterberry fruits (Fig. 3; Table 3). *Drosophila orena*, on the other hand, performs best on waterberry. One explanation for this would be that performance trade‐offs resulted in physiological specialization on waterberry before, during, or after *D. orena* evolved a behavioral preference for waterberry. An important caveat is that our measurement of performance does not allow us to differentiate between a female's propensity to lay eggs versus a decrease in egg hatchability or larval development. Given the numbers of offspring produced by all other species on the substrates we assayed, we suggest that the most likely explanation is that *D. orena*’s behavioral preference for waterberry extends to a female's propensity to lay eggs. Future work is needed to test this hypothesis and whether *D. orena*’s preference for waterberry has led to local adaptation and strict specialization.

### THE GENETIC BASIS OF HOST PREFERENCE

The fact that *D. orena* and *D. erecta* are sister species that show behavioral preferences for different substrates allowed us to ask whether the same olfactory genes show evidence of positive selection in both species. We find two general patterns: (1) enrichment in *ω* suggest that *Obp*s and *Csp*s have been under positive selection in both lineages (Fig. 4A) and (2) unlike enrichment at the level of gene family, the overlap in the specific genes with evidence of positive selection is not greater than we would expect by chance. These findings indicate that the evolution of host preferences might be predisposed to involve *Obp*s and *Csp*s; however, the specific genes underlying a bout of specialization can differ. Interestingly, estimates of *ω* for olfactory genes along the generalist *D. yakuba* lineage do not show enrichment for positive selection relative to the rest of the genome, and in the specialist species *D. santomea*, *Csp*s, *Or*s, and *Gr*s are all enriched for estimates of positive selection. A related study found that *Or*s and *Gr*s are evolving more rapidly (including being lost) in the specialist species *D. sechellia* and *D. erecta* than in related generalists (McBride and Arguello 2007); however these specialists also show elevated *K_a_*/*K_s_* across their genome. The analyses we conducted here indicate that *Or*s and *Gr*s do not show enriched *Ka/Ks* relative to the rest of the genome. Others have suggested that genes acting at the periphery of the olfactory system (such as *Obp*s and *Csp*s) evolve more rapidly than those acting in more central positions, potentially due to pleiotropic effects being less constraining for “peripheral” genes (Lavagnino et al. 2012). This hypothesis could explain the lower and correlated estimates of *ω* we observe for *Gr*s and *Or*s in *D. orena* and *D. erecta* (Fig. 4A).

Outside of protein coding substitutions, olfactory genes can evolve through changes in gene expression or copy number (i.e., duplication or loss). We did not test mechanisms other than protein coding substitutions along the *D. orena* lineage; however, the growing number of studies testing the genetic basis of olfactory preferences in insects suggest that these preferences can be controlled by a diverse genetic toolkit (e.g., *Gr*s, *Or*s, and *Obp*s in *Acyrthosiphon pisum* [Duvaux et al. 2015; Eyres et al. 2016]; an *Or* in *Aedes aegypti* [McBride et al. 2014]). Explicit tests of the relative role of protein‐changing substitution, changes in expression, or gene duplication are needed to help us better understand the dynamics of evolving host preferences.

While did not find statistical evidence for convergent positive selection within *Obp*s and *Csp*s, our analysis of *ω* in *D. orena* and *D. erecta* would not allow us to detect convergence due to changes in a small number (one or two) of amino acid residues or gene expression. Moreover, a lack of statistical convergence does not negate the possibility that individual loci are important for, and repeatedly used during, specialization onto different hosts. For example, *Obp99a*, *Obp99c*, and *Obp99d*, show signs of positive selection within *D. orena* and *D. erecta*, and mutations in different amino acid residues within these loci have been implicated in behavioral responses to acetophenone (*Obp99a* and *Obp99d*; Wang et al. 2010) and benzaldehyde in *D. melanogaster* (Wang et al. 2007). Two other *Obp*s with signatures of positive selection in *D. orena* – *Obp57d* and *Obp57e* – have been implicated in *D. sechellia*’s preference for the fruit of *Morinda citrifolia* (Matsuo et al. 2007). Notably, the complementation tests we conducted show that hemizygous individuals jointly expressing *Obp57a^ore^*, *Obp57b^ore^*, and *Obp57e^ore^* alleles have a slight preference for waterberry fruit (Fig. 4), suggesting that *Obp57e* is involved in host specialization in both *D. sechellia* and *D. orena*. We also find evidence for positive selection on *Obp93a* alleles in *D. orena* and *D. erecta*, a gene that is differentially expressed among population of *D. mojavensis* adapted to different species of cacti (Bono et al. 2011; Matzkin and Markow 2013). Finally, we find a signature of positive selection on *Obp83a* in *D. orena*, a locus that has previously been implicated in host seeking behavior in tsetse flies (Liu et al. 2012; Macharia et al. 2016). These individual examples could represent genes that are important for the evolution of different preference traits. It would be interesting to test this hypothesis by comparing these genes’ role in behavioral specialization across specialist and generalist species, spanning a large phylogenetic distance. These types of tests will add to our growing understanding of the genetic basis of adaptation, and the genetic toolkit deployed during specialization into different environments, a common evolutionary strategy.

Associate Editor: S. Wright

Handling Editor: J. Slate

## Supporting information


**Table S1**. Location of sites sampled on the island of Bioko.
**Table S2**. *Drosophila melanogaster* deficiency stocks used for complementation mapping.
**Table S3**. See attached Excel spreadsheet for information on additional genes spanned by the deficiencies used in this study.
**Table S4**. Distribution of the five species of the *melanogaster* species group found on Bioko along an altitudinal gradient. Counts are aggregates from three different trap substrate (bananas, mangoes, and waterberries).
**Table S5**. Loadings for the principle components analysis carried out on ‘bioclime’ variables sampled across the island of Bioko.
**Table S6**. Ka/Ks estimates for olfactory genes in the *D. orena* and *D. erecta* linages.
**Table S7**. Ka/Ks estimates for olfactory genes in the *D. yakuba* and *D. santomea* linages.
**Table S8**. Complementation mapping in mel/ore hybrids.

**Table‐S3**
