# Supplementary material for: A nonrandom subset of olfactory genes is associated with host preference in the fruit fly Drosophila orena
Source: Evol Lett. 2017 May 9;1(2):73–85. doi: 10.1002/evl3.7 (PMC6121841; doi:10.1002/evl3.7)
Supplement: Supplementary file 1 — Table S1. Location of sites sampled on the island of Bioko. Table S2. Drosophila melanogaster deficiency stocks used for complementation mapping. Table S3. See attached Excel spreadsheet for information on additional genes spanned by the deficiencies used in this study. Table S4. Distribution of the five species of the melanogaster species group found on Bioko along an altitudinal gradient. Counts are aggregates from three different trap substrate (bananas, mangoes, and waterberries). Table S5. Loadings for the principle components analysis carried out on ‘bioclime’ variables sampled across the island of Bioko. Table S6. Ka/Ks estimates for olfactory genes in the D. orena and D. erecta linages. Table S7. Ka/Ks estimates for olfactory genes in the D. yakuba and D. santomea linages. Table S8. Complementation mapping in mel/ore hybrids. [file EVL3-1-73-s001.docx]

**Supplemental Information.**

**I) Supplementary Methods.**

*Isofemale lines.*

Ninety-one *D. orena* females were not used in the experiments described above and were instead transferred to individual vials cornmeal food supplemented with boiled bananas to establish isofemale lines. The vast majority (85) of these females died without leaving progeny. Seven of these isofemale lines were successfully establish and transported to the USA (USDA permit: P526-15-02964). The successfully-established lines with their collection details are listed in Table S9.

*The climatic niche of* D. orena *on Bioko.*

We described variation in climate at each of our sampling locations and across the island of Bioko by extracting data from 19 contemporary bioclimatic variables from the Worldclim database (<http://www.worldclim.org/bioclim>). Bioclimatic variables were extracted for each sampling location and at each vertex of a 0.01degree grid covering the island (ranging from 8.37° to 9.02° longitude and 3.17° to 3.83° latitude) using the ‘raster’ library in R. We collapsed these data into 116 locations that differed from all other locations in at least 1 bioclimatic variable and carried out a principal components (PC) analysis using the *rda* function of the R library “vegan” (Oksanen *et al.* 2016). We then use this analysis to qualitatively summarize the climatic niche of *D. orena* on the island of Bioko.

D. orena *genome re-sequencing.*

We collected genome-wide sequence data from a single line of *D. orena* (Bioko1; Table S1). We extracted DNA from a single female fly using the QIAamp DNA Micro Kit (Qiagen, Chatsworth, CA, USA), following the manufacturer’s instruction. This protocol yields ~50 ng of DNA per fly per extraction. A library of genomic DNA was constructed using this extraction following the Kappa protocol for TrueSeq at the sequencing facility of the University of North Carolina, Chapel Hill. This library was then sequenced on a single lane of the HiSeq 2000 machine with v3.0 chemistry and 150 × 2 cycles.

Sequence reads were mapped to the *D. erecta* genome (Clark *et al.* 2007) using bwa version 0.7.12 (Li & Durbin 2009, 2010). Bam files were merged using Samtools version 0.1.19 (Li *et al.* 2009). Indels were identified and reads were locally remapped in the merged bam files using the RealignerTargetCreator and IndelRealigner tools available from the Broad Institute’s Genome Analysis Tool Kit (GATK) version 3.2-2 (McKenna *et al.* 2010; DePristo *et al.* 2011). SNP genotyping was done using GATK UnifiedGenotyper with the parameter het = 0.01. The following filters were applied to the resulting vcf file: QD = 2.0, FS_filter = 60.0, MQ_filter = 30.0, MQ_Rank_Sum_filter = -12.5, and Read_Pos_Rank_Sum_filter = -8.0. Ambiguous nucleotide characters were used to identify the two alleles at heterozygous sites. The resulted mean per-site coverage was 26.16X.

*Genome re-sequencing of the* D. yakuba *line used as an outgroup for Ka / Ks tests.*

We created a Nextera library for a single *D. yakuba* isofemale line derived from a female collected at Bom Sucesso on the island of São Tomé (line name: “1_19”) at the sequencing facility of the University of Illinois, Urbana-Champaign. DNA was segmented using Nextera kits which uses proprietary transposases to fragment DNA.

The *D. yakuba* library was sequenced on a single lane, obtaining paired 101bp reads. As with *D. orena*, we mapped these reads to the *D. erecta* reference genome. The resulting mean per-site coverage was 9.6X.

*Ka / Ks analysis of* D. santomea *and* D. yakuba.

In addition to our analysis of the ratio of synonymous (*K_s_*) to non-synonymous (*K_a_*) substitutions (*ω*) along the *D. orena* and *D. erecta* lineages, we computed *ω* for the related species *D. santomea* and *D. yakuba*. The clade ((*D. yakuba*, *D. santomea*), *D. teissieri*) is sister to the clade (*D. orena*, *D. erecta*). *Drosophila santomea* is commonly found in association with the fruit of *Ficus chlamydocarpa fernandesiana* suggesting that it has some preference for this fruit as a host (Lachaise *et al.* 1988; Cariou *et al.* 2001) while *D. yakuba* is a generalist species (David *et al.* 2007). We computed *ω* using re-sequencing data from 17 *D. santomea* isofemale lines and 56 *D. yakuba* using *D. teissieri* as the outgroup species. Sequences for each of these lines were generated as part of another study and are reported in Turissini and Matute (*In Revision*).

As with *D. orena* and *D. erecta* we focused our analysis on the ratio of derived non-synonymous to synonymous substitutions that were unique to either the *D. santomea* or *D. yakuba* lineage. For *D. santomea* we were able to estimate *ω* (i.e. the locus contained at least one synonymous substitution) for 10 *Obp*s, 12 *Csp*s, 35 *Or*s, 28 *Gr*s, and 6517 additional genes. For *D. yakuba* we were able to estimate *ω* for 8 *Obp*s, 6 *Csp*s, 30 *Or*s, 22 *Gr*s, and 5611 additional genes (Table S6). We compared the proportion of *Obp*, *Csp*, *Or*, and *Gr* loci with evidence of positive selection (i.e. *ω* ≥ 1) both among these different classes of olfactory loci and between each class of olfactory locus and the remaining genes for which we were able to estimate *ω* using Fisher’s Exact Tests (FETs).

**II) Supplementary Results.**

*The climatic niche of* D. orena *on Bioko.*

The lowest altitude that we collected *D. orena* from was 1,200 m above sea level and the highest was 2,020 m (Figures 1A and 2A; Table S1). The relative abundances of the species from the *melanogaster* subgroup we collected on Bioko varied across elevations (χ^2^ = 2,254.3; d.f. = 16; *P* < 1 × 10^-15^; Figure 2A; Table S3), and the relative abundance of *D. orena* was positively correlated with altitude (*ρ* = 0.975; S = 0.51; *P* = 0.005). In addition to only being found above 1,200 m, the sites where we collected *D. orena* encompass only 26.6% and 31.8% of the climatic variation on the island (as summarized by PCA; Figure 1A). These locations tend to be wet and cool relative to the rest of the island (Figure 1B; see table Table S4 for PC loadings). Anecdotally, the abundance of waterberry appeared to be correlated with the abundance of *D. orena*, leading us to hypothesize that *D. orena* might utilize waterberry fruits as a host.

**III) Supplementary References.**

Cariou ML, Silvain JF, Daubin V, Da Lage JL, Lachaise D (2001) Divergence between *Drosophila santomea* and allopatric or sympatric populations of *D. yakuba* using paralogous amylase genes and migration scenarios along the Cameroon volcanic line. *Molecular Ecology*, **10**, 649–660.

Clark AG, Eisen MB, Smith DR *et al.* (2007) Evolution of genes and genomes on the *Drosophila* phylogeny. *Nature*, **450**, 203–18.

David JR, Lemeunier F, Tsacas L, Yassin A (2007) The historical discovery of the nine species in the *Drosophila melanogaster* species subgroup. *Genetics*, **177**, 1969–1973.

DePristo MA, Banks E, Poplin R *et al.* (2011) A framework for variation discovery and genotyping using next-generation DNA sequencing data. *Nature Genetics*, **43**, 491–498.

Lachaise D, Cariou M-L, David JR *et al.* (1988) Historical Biogeography of the *Drosophila melanogaster* Species Subgroup. In: *Evolutionary Biology* (eds Hecht MK, Wallace B, Prance GT), pp. 159–225. Springer US, Boston, MA.

Li H, Durbin R (2009) Fast and accurate short read alignment with Burrows-Wheeler transform. *Bioinformatics*, **25**, 1754–1760.

Li H, Durbin R (2010) Fast and accurate long-read alignment with Burrows-Wheeler transform. *Bioinformatics*, **26**, 589–595.

Li H, Handsaker B, Wysoker A *et al.* (2009) The Sequence Alignment/Map format and SAMtools. *Bioinformatics*, **25**, 2078–2079.

McKenna A, Hanna M, Banks E *et al.* (2010) The Genome Analysis Toolkit: A MapReduce framework for analyzing next-generation DNA sequencing data. *Genome Research* , **20**, 1297–1303.

Oksanen J, Blanchet FG, Friendly M *et al.* (2016) vegan: Community Ecology Package.

**IV) Supplementary Tables**:

**Table S1.** Location of sites sampled on the island of Bioko.

| Latitude | Longitude | Altitude |
| --- | --- | --- |
| 3.352972 | 8.625111 | 2020 |
| 3.392778 | 8.656806 | 1650 |
| 3.403905 | 8.672271 | 1200 |
| 3.422422 | 8.646339 | 650 |
| 3.451556 | 8.616556 | 200 |

**Table S2.** *Drosophila melanogaster* deficiency stocks used for complementation mapping.

| **df stock #** | **cytological position of df** | **What’s this?** | **Obp(s) covered** |
| --- | --- | --- | --- |
| 25678 | 2R:19,632,024-2R:20,199,03 | 56E1--56F9 | *Obp56e, Obp56g, Obp56i* |
| 27354 | 2R:19,451,027-2R:19,501,804 | 56D8--56D14 | None |
| 26554 | 2R:20,424,117-2R:20,870,855 | 57A2--57B3 | *Obp57a, Obp57b, Obp57e* |
| 7552 | 2R:20,582,780-2R:20,836,033 | 57A6--57B3 | None |
| 7780 | 2L:1,989,057-2L:2,152,458..2,152,458 | 22B8--22D1 | *Obp22a* |
| 7779 | 2L:1,737,960-2L:2,010,136 | 22B2--22B8 | *Obp22a* |
| 7144 | 2L:2,175,620-2L:2,450,829 | 22D1--22F2 | None |
| 24968 | 3R:5,648,361-3R:6,211,946 | 83B7--83E1 | *Obp83cd* |
| 7778 | 2L:1,716,977-2L:1,909,976 | 22B1--22B5 | None |
| 24346 | 3R:5,716,821-3R:6,101,985 | 83C1--83D4 | *Obp83cd* |
| 24345 | 3R:5,716,821-3R:6,068,621 | 83C1--83D3 | None |
| 7443 | 3R:5,632,351-3R:5,861,298 | 83B7--83D1 | None |
| 7722 | X:20,330,532-X:20,468,383 | 19C4--19D1 | *Obp19b* |
| 25738 | X:19,788,713-X:20,379,977 | 18F2--19C5 | None |

**Table S3.** See attached Excel spreadsheet for information on additional genes spanned by the deficiencies used in this study.

**Table S4.** Distribution of the five species of the *melanogaster* species group found on Bioko along an altitudinal gradient. Counts are aggregates from three different trap substrate (bananas, mangoes, and waterberries).

| **Altitude** | ***D. melanogaster*** | ***D. simulans*** | ***D. teissieri*** | ***D. yakuba*** | ***D. orena*** |
| --- | --- | --- | --- | --- | --- |
| 200 | 465 | 52 | 0 | 164 | 0 |
| 650 | 804 | 360 | 5 | 237 | 0 |
| 1,200 | 402 | 179 | 425 | 191 | 22 |
| 1,650 | 200 | 318 | 300 | 167 | 74 |
| 2,020 | 4 | 29 | 50 | 32 | 97 |

**Table S5.** Loadings for the principle components analysis carried out on ‘bioclime’ variables sampled across the island of Bioko.

| BIOClim | PC1 | PC2 | PC3 | PC4 | PC5 |
| --- | --- | --- | --- | --- | --- |
| bio1 | 0.2041 | 0.1034 | 0.2390 | -0.0044 | 0.0556 |
| bio2 | -0.0061 | -0.0152 | -0.0032 | 0.0281 | -0.0116 |
| bio3 | 0.0004 | -0.0044 | 0.0042 | 0.0034 | -0.0005 |
| bio4 | -0.1603 | -0.0217 | -0.0669 | 0.1985 | -0.7719 |
| bio5 | 0.2054 | 0.0931 | 0.2448 | 0.0218 | 0.0373 |
| bio6 | 0.2136 | 0.1074 | 0.2553 | -0.0120 | 0.0546 |
| bio7 | -0.0081 | -0.0142 | -0.0105 | 0.0338 | -0.0173 |
| bio8 | 0.2035 | 0.1055 | 0.2313 | -0.0194 | 0.0596 |
| bio9 | 0.2029 | 0.1025 | 0.2354 | -0.0024 | 0.0486 |
| bio10 | 0.2035 | 0.1032 | 0.2390 | -0.0027 | 0.0380 |
| bio11 | 0.2068 | 0.1033 | 0.2381 | -0.0045 | 0.0664 |
| bio12 | -0.6804 | 0.0384 | 0.5891 | -0.4300 | -0.0433 |
| bio13 | -0.1487 | 0.2035 | -0.2556 | -0.1038 | 0.1413 |
| bio14 | -0.0184 | -0.0419 | 0.0116 | 0.0477 | 0.1190 |
| bio15 | 0.0084 | 0.0429 | -0.0241 | -0.0145 | -0.0128 |
| bio16 | -0.3220 | 0.5074 | -0.2761 | 0.0919 | 0.3746 |
| bio17 | -0.1139 | -0.1435 | -0.0221 | 0.1002 | 0.3481 |
| bio18 | -0.2596 | -0.4254 | 0.2697 | 0.7080 | 0.2331 |
| bio19 | -0.0920 | 0.6479 | 0.1671 | 0.4896 | -0.1804 |

**Table S6.** Ka/Ks estimates for genes controlling odorant binding proteins along the *D. orena* and *D. erecta* linages.

| FB_erecta | class | Ka/Ks _erecta | Ka/Ks _orena | Ka_orena_derived | Ka_erecta_derived | Ks_orena_derived | Ks_erecta_derived |
| --- | --- | --- | --- | --- | --- | --- | --- |
| FBgn0116874 | Gr | 14 | NA | 7 | 14 | 0 | 1 |
| FBgn0114181 | Obp | NA | 9 | 9 | 2 | 1 | 0 |
| FBgn0108030 | Csp | 2 | 2.3333 | 7 | 2 | 3 | 1 |
| FBgn0114174 | Obp | NA | 3 | 9 | 10 | 3 | 0 |
| FBgn0116296 | Gr | 4 | 3.5 | 7 | 8 | 2 | 2 |
| FBgn0103091 | Csp | 4.5 | 12 | 12 | 18 | 1 | 4 |
| FBgn0109473 | Csp | NA | NA | 0 | 0 | 0 | 0 |
| FBgn0116240 | Csp | 1 | 3.75 | 15 | 2 | 4 | 2 |
| FBgn0107820 | Obp | NA | 1.5 | 3 | 2 | 2 | 0 |
| FBgn0116763 | Csp | NA | NA | 0 | 0 | 0 | 0 |
| FBgn0109697 | Csp | 2 | 1.4 | 7 | 4 | 5 | 2 |
| FBgn0110230 | Or | 4.3333 | 1.6667 | 10 | 13 | 6 | 3 |
| FBgn0113724 | Csp | 4 | 2.4 | 12 | 12 | 5 | 3 |
| FBgn0105381 | Obp | 2.6 | 3 | 18 | 13 | 6 | 5 |
| FBgn0113261 | Gr | 2.3333 | 1.75 | 14 | 14 | 8 | 6 |
| FBgn0115386 | Csp | NA | 1.6667 | 5 | 4 | 3 | 0 |
| FBgn0106280 | Or | 3 | 2 | 14 | 18 | 7 | 6 |
| FBgn0103090 | Csp | 1.5714 | 4.5 | 9 | 11 | 2 | 7 |
| FBgn0113073 | Obp | 1.6667 | 2 | 4 | 5 | 2 | 3 |
| FBgn0113071 | Obp | 0.6667 | 2.5 | 5 | 2 | 2 | 3 |
| FBgn0103910 | Csp | 2 | 2.1429 | 15 | 8 | 7 | 4 |
| FBgn0110210 | Obp | 2 | 1.5 | 3 | 4 | 2 | 2 |
| FBgn0111492 | Obp | 1.8 | 2 | 4 | 9 | 2 | 5 |
| FBgn0103972 | Obp | 5 | 1 | 3 | 5 | 3 | 1 |
| FBgn0112797 | Csp | 1 | 2.5 | 5 | 1 | 2 | 1 |
| FBgn0107104 | Obp | 2.3333 | 1.3333 | 4 | 7 | 3 | 3 |
| FBgn0114092 | Csp | NA | 1 | 2 | 4 | 2 | 0 |
| FBgn0116694 | Obp | 2 | 3.5 | 7 | 4 | 2 | 2 |
| FBgn0103977 | Obp | 1.6667 | 2.5 | 5 | 5 | 2 | 3 |
| FBgn0103006 | Or | 3.3333 | 0.8 | 8 | 10 | 10 | 3 |
| FBgn0114175 | Obp | 1 | 1 | 3 | 1 | 3 | 1 |
| FBgn0104300 | Obp | 2.5 | 1 | 4 | 5 | 4 | 2 |
| FBgn0112220 | Gr | 1.5714 | 1.1667 | 7 | 11 | 6 | 7 |
| FBgn0114985 | Gr | 2.2 | 1.1 | 11 | 11 | 10 | 5 |
| FBgn0113687 | Gr | 1 | 1.7143 | 12 | 4 | 7 | 4 |
| FBgn0113725 | Csp | 0.7778 | 7 | 14 | 7 | 2 | 9 |
| FBgn0114210 | Obp | 0.4286 | 4 | 8 | 3 | 2 | 7 |
| FBgn0114209 | Obp | 1 | 2 | 2 | 1 | 1 | 1 |
| FBgn0116525 | Or | 0.5 | 3 | 3 | 4 | 1 | 8 |
| FBgn0117408 | Csp | NA | NA | 0 | 0 | 0 | 0 |
| FBgn0114597 | Obp | 1.2 | 1.75 | 7 | 6 | 4 | 5 |
| FBgn0109670 | Or | 0.5714 | 1.55 | 31 | 8 | 20 | 14 |
| FBgn0111493 | Obp | 1.6667 | 0.6667 | 2 | 5 | 3 | 3 |
| FBgn0112639 | Obp | 0.6667 | 1 | 5 | 4 | 5 | 6 |
| FBgn0113263 | Gr | 1.2857 | 2 | 6 | 9 | 3 | 7 |
| FBgn0105561 | Gr | 1.5455 | 0.5714 | 8 | 17 | 14 | 11 |
| FBgn0103152 | Or | 2 | 0.4444 | 4 | 12 | 9 | 6 |
| FBgn0115020 | Gr | 1 | 1 | 15 | 9 | 15 | 9 |
| FBgn0102885 | Obp | 1 | 0.7857 | 11 | 9 | 14 | 9 |
| FBgn0109539 | Gr | 0.9048 | 1.0769 | 14 | 19 | 13 | 21 |
| FBgn0111823 | Csp | 4 | 0.6667 | 4 | 4 | 6 | 1 |
| FBgn0110514 | Obp | 0.5 | 1.25 | 5 | 4 | 4 | 8 |
| FBgn0112227 | Or | 0.7368 | 1.4545 | 16 | 14 | 11 | 19 |
| FBgn0112265 | Gr | 0.9 | 1.2727 | 14 | 9 | 11 | 10 |
| FBgn0113076 | Obp | 0.2857 | 1 | 5 | 2 | 5 | 7 |
| FBgn0115672 | Obp | 1 | 0 | 0 | 3 | 1 | 3 |
| FBgn0117344 | Obp | 2.5 | 0.6667 | 4 | 5 | 6 | 2 |
| FBgn0112482 | Or | 0.8 | 0.4286 | 6 | 8 | 14 | 10 |
| FBgn0112264 | Gr | 0.75 | 0.6818 | 15 | 9 | 22 | 12 |
| FBgn0109666 | Or | 0.6667 | 1 | 7 | 4 | 7 | 6 |
| FBgn0114211 | Obp | 0.3333 | 2.5 | 5 | 2 | 2 | 6 |
| FBgn0105199 | Or | 0.5455 | 0.8182 | 9 | 6 | 11 | 11 |
| FBgn0114949 | Obp | 1 | 0.8333 | 5 | 3 | 6 | 3 |
| FBgn0114727 | Obp | 0.4 | 1.5 | 6 | 2 | 4 | 5 |
| FBgn0106055 | Or | 0.56 | 1.087 | 25 | 14 | 23 | 25 |
| FBgn0116280 | Or | 0.7059 | 1.2857 | 9 | 12 | 7 | 17 |
| FBgn0112919 | Gr | 0.7778 | 0.9 | 9 | 7 | 10 | 9 |
| FBgn0112640 | Obp | 1.3333 | 0.1667 | 1 | 8 | 6 | 6 |
| FBgn0112389 | Gr | 1.6 | 0.375 | 3 | 8 | 8 | 5 |
| FBgn0115580 | Or | 0.7895 | 0.9 | 9 | 15 | 10 | 19 |
| FBgn0263823 | Obp | 0 | 2 | 2 | 0 | 1 | 2 |
| FBgn0109057 | Gr | 0.8333 | 0.4667 | 7 | 15 | 15 | 18 |
| FBgn0112355 | Obp | 1 | 0.6 | 3 | 4 | 5 | 4 |
| FBgn0112230 | Or | 0.5 | 1.3333 | 8 | 5 | 6 | 10 |
| FBgn0114327 | Gr | 0.8182 | 1.125 | 9 | 9 | 8 | 11 |
| FBgn0103857 | Gr | 0.9286 | 0.75 | 9 | 13 | 12 | 14 |
| FBgn0112641 | Obp | 1.5 | 0.5 | 2 | 6 | 4 | 4 |
| FBgn0103911 | Csp | 1.5 | 0.5556 | 5 | 6 | 9 | 4 |
| FBgn0116998 | Or | 0.5455 | 1.3333 | 8 | 6 | 6 | 11 |
| FBgn0103068 | Or | 0.4444 | 1.4286 | 10 | 4 | 7 | 9 |
| FBgn0103333 | Or | 0.2222 | 0.875 | 7 | 4 | 8 | 18 |
| FBgn0116942 | Gr | 0.8 | 0.6429 | 9 | 8 | 14 | 10 |
| FBgn0104451 | Gr | 0.8125 | 0.5714 | 4 | 13 | 7 | 16 |
| FBgn0115885 | Or | 3 | 0.3077 | 4 | 6 | 13 | 2 |
| FBgn0107594 | Or | 0.2308 | 0.5 | 12 | 3 | 24 | 13 |
| FBgn0110556 | Gr | 0.5833 | 0.65 | 13 | 7 | 20 | 12 |
| FBgn0103858 | Gr | 0.75 | 0.4286 | 3 | 12 | 7 | 16 |
| FBgn0106996 | Gr | 0.5385 | 0.7 | 7 | 7 | 10 | 13 |
| FBgn0111847 | Csp | 0.25 | 0.4 | 2 | 1 | 5 | 4 |
| FBgn0113074 | Obp | 0.625 | 1.25 | 5 | 5 | 4 | 8 |
| FBgn0116941 | Gr | 0.5 | 1 | 8 | 5 | 8 | 10 |
| FBgn0107146 | Or | 0.3571 | 0.6154 | 8 | 5 | 13 | 14 |
| FBgn0103475 | Or | 0.8095 | 0.375 | 3 | 17 | 8 | 21 |
| FBgn0110519 | Gr | 0.375 | 1.0833 | 13 | 6 | 12 | 16 |
| FBgn0114951 | Obp | NA | 0.5 | 4 | 4 | 8 | 0 |
| FBgn0111065 | Gr | 0.2667 | 0.8889 | 8 | 4 | 9 | 15 |
| FBgn0117392 | Obp | 0.75 | 0.6 | 3 | 3 | 5 | 4 |
| FBgn0103894 | Or | 0.625 | 0.6364 | 7 | 5 | 11 | 8 |
| FBgn0116940 | Gr | 0.5 | 0.5 | 10 | 7 | 20 | 14 |
| FBgn0103717 | Gr | 0.6667 | 0.3077 | 4 | 4 | 13 | 6 |
| FBgn0116708 | Gr | 0.2222 | 0.5 | 7 | 2 | 14 | 9 |
| FBgn0115886 | Or | 0.4375 | 0.8 | 12 | 7 | 15 | 16 |
| FBgn0115884 | Or | 0.25 | 0.75 | 9 | 4 | 12 | 16 |
| FBgn0107348 | Gr | 0.7 | 0.6364 | 7 | 7 | 11 | 10 |
| FBgn0110982 | Gr | 0.4167 | 1.375 | 11 | 5 | 8 | 12 |
| FBgn0103856 | Gr | 0.5556 | 0.6 | 6 | 10 | 10 | 18 |
| FBgn0103476 | Or | 0.7391 | 0.4118 | 7 | 17 | 17 | 23 |
| FBgn0114831 | Or | 0.8235 | 0.3 | 3 | 14 | 10 | 17 |
| FBgn0114642 | Gr | 0.3846 | 0.6 | 9 | 5 | 15 | 13 |
| FBgn0103339 | Or | 0.5263 | 0.5 | 5 | 10 | 10 | 19 |
| FBgn0113021 | Gr | 0.5833 | 0.5556 | 5 | 7 | 9 | 12 |
| FBgn0064622 | Gr | 0.5714 | 0.5 | 11 | 8 | 22 | 14 |
| FBgn0107436 | Gr | 0.75 | 0.1538 | 2 | 12 | 13 | 16 |
| FBgn0114950 | Obp | 0.3333 | 0.6 | 3 | 3 | 5 | 9 |
| FBgn0116957 | Or | 0.5 | 1 | 3 | 5 | 3 | 10 |
| FBgn0107775 | Gr | 0.6667 | 0.5556 | 5 | 10 | 9 | 15 |
| FBgn0068847 | Obp | 0.4 | 1 | 1 | 2 | 1 | 5 |
| FBgn0114326 | Gr | 0.5 | 0.5556 | 5 | 3 | 9 | 6 |
| FBgn0114600 | Obp | 0.5 | 0.1667 | 1 | 4 | 6 | 8 |
| FBgn0116705 | Gr | 0.3333 | 0.7778 | 7 | 5 | 9 | 15 |
| FBgn0103852 | Gr | 0.5 | 0.619 | 13 | 8 | 21 | 16 |
| FBgn0109066 | Or | 0.2941 | 0.7273 | 8 | 5 | 11 | 17 |
| FBgn0116223 | Or | 0.7778 | 0.3636 | 4 | 7 | 11 | 9 |
| FBgn0106240 | Or | 0.4667 | 0.5294 | 9 | 7 | 17 | 15 |
| FBgn0113473 | Gr | 0.75 | 0.3636 | 4 | 3 | 11 | 4 |
| FBgn0117263 | Or | 0.2778 | 0.7143 | 10 | 5 | 14 | 18 |
| FBgn0111063 | Or | 0.2 | 0.4583 | 11 | 4 | 24 | 20 |
| FBgn0104533 | Or | 0.25 | 1.3333 | 8 | 7 | 6 | 28 |
| FBgn0111491 | Obp | 0.125 | 1 | 1 | 1 | 1 | 8 |
| FBgn0107431 | Gr | 0.625 | 0.2353 | 4 | 10 | 17 | 16 |
| FBgn0103483 | Gr | 0.7143 | 0.25 | 4 | 10 | 16 | 14 |
| FBgn0108063 | Or | 0.4 | 0.6 | 6 | 6 | 10 | 15 |
| FBgn0102919 | Or | 0.6923 | 0.2963 | 8 | 9 | 27 | 13 |
| FBgn0107593 | Or | 0.2 | 0.4118 | 7 | 1 | 17 | 5 |
| FBgn0064621 | Gr | 0.2941 | 0.55 | 11 | 5 | 20 | 17 |
| FBgn0105062 | Or | 1.375 | 0.2826 | 13 | 11 | 46 | 8 |
| FBgn0107636 | Or | 0.2 | 0.1 | 1 | 2 | 10 | 10 |
| FBgn0105922 | Csp | 0 | 0.875 | 7 | 0 | 8 | 12 |
| FBgn0113072 | Or | 0.5 | 0.5 | 4 | 7 | 8 | 14 |
| FBgn0111126 | Or | 0.1333 | 0.5 | 6 | 2 | 12 | 15 |
| FBgn0108771 | Or | 0.3333 | 0.5385 | 7 | 4 | 13 | 12 |
| FBgn0114834 | Obp | 0.4 | 0.3333 | 2 | 2 | 6 | 5 |
| FBgn0117453 | Or | 0.25 | 0.4375 | 7 | 5 | 16 | 20 |
| FBgn0115848 | Gr | 0.3 | 0.4167 | 5 | 3 | 12 | 10 |
| FBgn0103385 | Gr | 0.2 | 0.4737 | 9 | 4 | 19 | 20 |
| FBgn0110197 | Or | 0.1667 | 0.1875 | 3 | 2 | 16 | 12 |
| FBgn0110155 | Or | 0.375 | 0.2143 | 3 | 9 | 14 | 24 |
| FBgn0117003 | Gr | 0.2353 | 0.5833 | 7 | 4 | 12 | 17 |
| FBgn0104844 | Gr | 0.2778 | 0.2692 | 7 | 5 | 26 | 18 |
| FBgn0116333 | Or | 0.4444 | 0.2353 | 4 | 4 | 17 | 9 |
| FBgn0105858 | Obp | 0.5 | 0 | 0 | 4 | 7 | 8 |
| FBgn0112292 | Obp | 0 | 0.5 | 4 | 0 | 8 | 6 |
| FBgn0114172 | Obp | 0 | 0.3333 | 1 | 0 | 3 | 4 |
| FBgn0114180 | Obp | 0.6667 | 0 | 0 | 2 | 1 | 3 |
| FBgn0068848 | Obp | 1.3333 | 0.0909 | 1 | 4 | 11 | 3 |
| FBgn0111064 | Gr | 0.125 | 0.4 | 6 | 3 | 15 | 24 |
| FBgn0107435 | Gr | 0.2632 | 0.2143 | 3 | 5 | 14 | 19 |
| FBgn0107437 | Gr | 0.1 | 0.1818 | 2 | 1 | 11 | 10 |
| FBgn0105131 | Gr | 0.4 | 0.1429 | 2 | 4 | 14 | 10 |
| FBgn0112375 | Or | 0.2 | 0.3333 | 6 | 6 | 18 | 30 |
| FBgn0109672 | Or | 0.2 | 0.3182 | 7 | 4 | 22 | 20 |
| FBgn0107434 | Gr | 0.25 | 0.1765 | 3 | 5 | 17 | 20 |
| FBgn0115911 | Gr | 0.0625 | 0.4 | 4 | 1 | 10 | 16 |
| FBgn0107592 | Or | 0.1875 | 0.1429 | 2 | 3 | 14 | 16 |
| FBgn0112531 | Or | 0.1667 | 0.1333 | 2 | 2 | 15 | 12 |
| FBgn0103062 | Gr | 0.2 | 0 | 0 | 3 | 5 | 15 |
| FBgn0107682 | Or | 0 | 0.1852 | 5 | 0 | 27 | 11 |
| FBgn0116062 | Or | 0.2222 | 0.0385 | 1 | 2 | 26 | 9 |
| FBgn0116927 | Or | 0.0625 | 0.1111 | 3 | 1 | 27 | 16 |
| FBgn0106504 | Gr | 0 | 0.1538 | 4 | 0 | 26 | 15 |
| FBgn0103151 | Or | 0.25 | 0.0909 | 1 | 2 | 11 | 8 |
| FBgn0107432 | Gr | 0.1111 | 0 | 0 | 2 | 12 | 18 |
| FBgn0112228 | Or | 0.1429 | 0 | 0 | 3 | 11 | 21 |
| FBgn0115677 | Gr | 0.1818 | 0 | 0 | 4 | 34 | 22 |
| FBgn0115676 | Gr | 0.1364 | 0.0435 | 1 | 3 | 23 | 22 |
| FBgn0103719 | Gr | 0.0909 | 0 | 0 | 1 | 11 | 11 |
| FBgn0110521 | Or | 0 | 0.1667 | 1 | 0 | 6 | 13 |
| FBgn0102979 | Obp | 0 | 0 | 0 | 0 | 3 | 2 |
| FBgn0105402 | Or | NA | 0 | 0 | 0 | 6 | 0 |
| FBgn0109673 | Obp | 0 | 0 | 0 | 0 | 9 | 3 |
| FBgn0105278 | Or | NA | NA | 0 | 0 | 0 | 0 |
| FBgn0105279 | Or | NA | NA | 0 | 0 | 0 | 0 |
| FBgn0105401 | Or | NA | NA | 0 | 0 | 0 | 0 |

**Table S7.** Ka/Ks estimates for olfactory genes in the *D. yakuba* and *D. santomea* linages.

| FB | class | Ka/Ks _yakuba | Ka/Ks _santomea | Ka _yak_derived | Ka _san_derived | Ka _both_derived | Ks _yak_derived | Ks _san_derived | Ks _both_derived |
| --- | --- | --- | --- | --- | --- | --- | --- | --- | --- |
| FBgn0234814 | Or | NA | 13 | 6 | 26 | 3 | 0 | 2 | 0 |
| FBgn0236623 | Or | NA | 5 | 1 | 5 | 0 | 0 | 1 | 0 |
| FBgn0229586 | Csp | NA | 4 | 0 | 4 | 0 | 0 | 1 | 0 |
| FBgn0240955 | Csp | 3 | NA | 3 | 2 | 0 | 1 | 0 | 0 |
| FBgn0229403 | Gr | NA | 3.5 | 0 | 7 | 1 | 0 | 2 | 0 |
| FBgn0229404 | Gr | 2 | 5 | 2 | 5 | 0 | 1 | 1 | 0 |
| FBgn0240785 | Gr | NA | 2 | 2 | 2 | 0 | 0 | 1 | 0 |
| FBgn0236739 | Or | 3 | 3 | 3 | 3 | 1 | 1 | 1 | 0 |
| FBgn0240901 | Gr | NA | 2.6667 | 1 | 8 | 0 | 0 | 3 | 0 |
| FBgn0229766 | Csp | NA | 3 | 0 | 3 | 0 | 0 | 1 | 0 |
| FBgn0229992 | Gr | NA | 2 | 0 | 2 | 0 | 0 | 1 | 0 |
| FBgn0230238 | Or | 0 | NA | 0 | 3 | 0 | 1 | 0 | 0 |
| FBgn0235603 | Gr | 0 | NA | 0 | 3 | 0 | 1 | 0 | 0 |
| FBgn0236637 | Or | 1 | 5 | 1 | 5 | 0 | 1 | 1 | 0 |
| FBgn0238813 | Or | 3.5 | 2 | 7 | 2 | 0 | 2 | 1 | 0 |
| FBgn0241477 | Csp | NA | 2 | 2 | 4 | 0 | 0 | 2 | 0 |
| FBgn0243298 | Csp | 5 | 1 | 5 | 1 | 0 | 1 | 1 | 0 |
| FBgn0230508 | Gr | 1.5 | 4 | 3 | 8 | 0 | 2 | 2 | 0 |
| FBgn0242794 | Csp | 0.6667 | 7 | 2 | 7 | 0 | 3 | 1 | 0 |
| FBgn0242993 | Or | 0.5 | 3 | 1 | 9 | 0 | 2 | 3 | 0 |
| FBgn0235959 | Or | 0.5 | 3.5 | 1 | 14 | 0 | 2 | 4 | 0 |
| FBgn0228215 | Gr | 1.6 | 2 | 8 | 4 | 1 | 5 | 2 | 0 |
| FBgn0229587 | Csp | NA | 1 | 1 | 1 | 0 | 0 | 1 | 0 |
| FBgn0230023 | Obp | NA | 1 | 1 | 1 | 0 | 0 | 1 | 0 |
| FBgn0234773 | Gr | 0.5 | NA | 1 | 4 | 0 | 2 | 0 | 0 |
| FBgn0236703 | Gr | 1 | NA | 3 | 1 | 1 | 3 | 0 | 0 |
| FBgn0238140 | Or | NA | 0 | 1 | 0 | 0 | 0 | 1 | 0 |
| FBgn0238657 | Gr | 0 | NA | 0 | 4 | 0 | 1 | 0 | 0 |
| FBgn0238663 | Gr | NA | 2 | 0 | 2 | 0 | 0 | 1 | 0 |
| FBgn0240922 | Or | 2 | 2 | 2 | 6 | 0 | 1 | 3 | 0 |
| FBgn0241221 | Gr | NA | NA | 0 | 2 | 0 | 0 | 0 | 0 |
| FBgn0241968 | Csp | 2 | 1.5 | 2 | 3 | 1 | 1 | 2 | 0 |
| FBgn0242366 | Or | 5 | 0 | 5 | 0 | 1 | 1 | 3 | 0 |
| FBgn0242933 | Or | 4 | 1.5 | 4 | 3 | 0 | 1 | 2 | 0 |
| FBgn0240941 | Or | NA | 0.6667 | 2 | 2 | 0 | 0 | 3 | 0 |
| FBgn0068111 | Obp | 2 | 1 | 2 | 1 | 0 | 1 | 1 | 0 |
| FBgn0230570 | Obp | 2 | NA | 2 | 1 | 0 | 1 | 0 | 0 |
| FBgn0231427 | Gr | NA | 1 | 1 | 2 | 0 | 0 | 2 | 0 |
| FBgn0231477 | Or | 0 | 4 | 0 | 4 | 0 | 2 | 1 | 0 |
| FBgn0233010 | Gr | 1.5 | 0 | 3 | 0 | 0 | 2 | 1 | 0 |
| FBgn0241222 | Gr | NA | 2 | 0 | 4 | 0 | 0 | 2 | 0 |
| FBgn0236689 | Or | 1.5 | 1.5 | 3 | 3 | 0 | 2 | 2 | 1 |
| FBgn0228210 | Or | NA | 1 | 0 | 1 | 0 | 0 | 1 | 0 |
| FBgn0229357 | Gr | 0.5 | 2 | 1 | 2 | 0 | 2 | 1 | 0 |
| FBgn0229365 | Or | 0 | NA | 0 | 1 | 0 | 1 | 0 | 0 |
| FBgn0229847 | Obp | NA | 1 | 0 | 1 | 0 | 0 | 1 | 0 |
| FBgn0230698 | Gr | 0 | 2 | 0 | 2 | 0 | 1 | 1 | 0 |
| FBgn0230731 | Obp | 1 | 1 | 1 | 1 | 0 | 1 | 1 | 0 |
| FBgn0231203 | Csp | 0 | 1.5 | 0 | 3 | 0 | 1 | 2 | 0 |
| FBgn0233791 | Or | 0.3333 | 1.6667 | 1 | 5 | 0 | 3 | 3 | 0 |
| FBgn0234772 | Gr | 0.3333 | 3 | 1 | 3 | 0 | 3 | 1 | 0 |
| FBgn0235079 | Or | 0 | 1.5 | 0 | 3 | 0 | 1 | 2 | 0 |
| FBgn0235925 | Gr | NA | 1 | 0 | 1 | 0 | 0 | 1 | 0 |
| FBgn0237595 | Or | NA | 1 | 0 | 1 | 0 | 0 | 1 | 0 |
| FBgn0238905 | Obp | 1 | NA | 1 | 0 | 0 | 1 | 0 | 0 |
| FBgn0239140 | Obp | NA | 1 | 0 | 1 | 0 | 0 | 1 | 0 |
| FBgn0241019 | Obp | NA | 0 | 0 | 0 | 0 | 0 | 1 | 0 |
| FBgn0241223 | Gr | 0 | NA | 0 | 1 | 0 | 1 | 0 | 0 |
| FBgn0241820 | Gr | 1.5 | 0.5 | 3 | 1 | 0 | 2 | 2 | 0 |
| FBgn0241940 | Or | 0.5 | NA | 2 | 2 | 0 | 4 | 0 | 0 |
| FBgn0241945 | Or | 1 | 1 | 1 | 4 | 0 | 1 | 4 | 0 |
| FBgn0242713 | Gr | 1 | NA | 1 | 1 | 0 | 1 | 0 | 0 |
| FBgn0243184 | Gr | NA | 0 | 1 | 0 | 0 | 0 | 1 | 0 |
| FBgn0230507 | Gr | 0.5 | 1 | 1 | 3 | 0 | 2 | 3 | 0 |
| FBgn0237409 | Or | 0.2 | 1.7143 | 1 | 12 | 0 | 5 | 7 | 0 |
| FBgn0230626 | Gr | 0.5 | 1.3333 | 2 | 4 | 0 | 4 | 3 | 1 |
| FBgn0235269 | Gr | 0 | 1 | 0 | 3 | 0 | 1 | 3 | 0 |
| FBgn0239088 | Gr | NA | 1 | 0 | 2 | 0 | 0 | 2 | 0 |
| FBgn0240718 | Or | 0 | 1 | 0 | 2 | 0 | 1 | 2 | 0 |
| FBgn0240921 | Or | 1 | 0.6667 | 1 | 2 | 0 | 1 | 3 | 1 |
| FBgn0241946 | Or | 0.25 | 1 | 1 | 1 | 1 | 4 | 1 | 0 |
| FBgn0230067 | Obp | 1 | 0 | 1 | 0 | 0 | 1 | 1 | 0 |
| FBgn0230586 | Or | 1 | 0 | 2 | 0 | 0 | 2 | 1 | 0 |
| FBgn0234719 | Or | 1 | 0.3333 | 2 | 1 | 0 | 2 | 3 | 1 |
| FBgn0234771 | Or | NA | 1 | 0 | 1 | 0 | 0 | 1 | 0 |
| FBgn0236649 | Csp | 0.5 | 0 | 1 | 0 | 1 | 2 | 2 | 0 |
| FBgn0230728 | Or | 0.3333 | 0.5 | 1 | 1 | 0 | 3 | 2 | 0 |
| FBgn0232367 | Or | 0.6667 | 0.4 | 2 | 2 | 0 | 3 | 5 | 1 |
| FBgn0233943 | Gr | 0.1429 | 1 | 1 | 2 | 0 | 7 | 2 | 0 |
| FBgn0239392 | Or | NA | 0.5 | 0 | 1 | 0 | 0 | 2 | 0 |
| FBgn0241467 | Csp | NA | 0.3333 | 0 | 1 | 0 | 0 | 3 | 0 |
| FBgn0242475 | Or | 0.25 | 0.25 | 1 | 1 | 1 | 4 | 4 | 1 |
| FBgn0242919 | Or | 0.3333 | NA | 1 | 0 | 0 | 3 | 0 | 0 |
| FBgn0234113 | Or | 0.5 | 0.2 | 1 | 1 | 0 | 2 | 5 | 0 |
| FBgn0242727 | Or | 0.25 | NA | 1 | 0 | 0 | 4 | 0 | 0 |
| FBgn0234540 | Gr | 0 | 0.1667 | 0 | 1 | 0 | 3 | 6 | 0 |
| FBgn0242563 | Gr | 0.2 | 0 | 1 | 0 | 0 | 5 | 7 | 0 |
| FBgn0068108 | Obp | 0 | 0 | 0 | 0 | 0 | 1 | 1 | 0 |
| FBgn0228458 | Gr | NA | 0 | 0 | 0 | 0 | 0 | 1 | 0 |
| FBgn0229627 | Obp | 0 | NA | 0 | 0 | 0 | 2 | 0 | 0 |
| FBgn0231204 | Csp | NA | 0 | 0 | 0 | 0 | 0 | 1 | 0 |
| FBgn0231646 | Obp | NA | 0 | 0 | 0 | 0 | 0 | 2 | 0 |
| FBgn0231915 | Gr | NA | 0 | 0 | 0 | 0 | 0 | 1 | 0 |
| FBgn0232970 | Or | 0 | 0 | 0 | 0 | 0 | 1 | 2 | 0 |
| FBgn0235228 | Or | NA | 0 | 0 | 0 | 0 | 0 | 1 | 0 |
| FBgn0236783 | Or | 0 | 0 | 0 | 0 | 0 | 2 | 4 | 0 |
| FBgn0237220 | Obp | NA | NA | 0 | 0 | 0 | 0 | 0 | 0 |
| FBgn0238493 | Or | NA | 0 | 0 | 0 | 0 | 0 | 1 | 0 |
| FBgn0238582 | Gr | 0 | NA | 0 | 0 | 0 | 1 | 0 | 0 |
| FBgn0238662 | Gr | 0 | NA | 0 | 0 | 0 | 1 | 0 | 0 |
| FBgn0240956 | Csp | NA | 0 | 0 | 0 | 0 | 0 | 1 | 0 |
| FBgn0241231 | Gr | NA | 0 | 0 | 0 | 0 | 0 | 1 | 0 |
| FBgn0241350 | Gr | NA | 0 | 0 | 0 | 0 | 0 | 1 | 0 |
| FBgn0242102 | Obp | 0 | 0 | 0 | 0 | 0 | 3 | 1 | 0 |

**Table S8.** Complementation mapping in mel/ore hybrids. Data are the number of individuals choosing cornmeal, remaining in the center vial (empty) or choosing waterberry, in ‘Y-maze’ type food choice assays. Odds-ratios were calculated as the number of flies carrying the deficiency that chose corn meal over the number of flies carrying the balancer that chose corn meal divided by the number of flies carrying the deficiency and choosing waterberry over the number carrying the balancer and choosing waterberry. P-values were calculated with the null hypothesis of 'less-than'.

| **Genotype** | **Deleted Obp** | **chromosomes** | **Corn meal** | **Empty** | **Water-berry** | **P** | **Odds-ratio** |
| --- | --- | --- | --- | --- | --- | --- | --- |
| ***melanogaster*** | **-** | **-** | **319** | **20** | **136** | **-** | **-** |
| ***orena*** | - | - | 58 | 38 | 374 | - | - |
| ***mel/ore*** | - | - | 26 | 20 | 4 | - | - |
| **25678** | *Obp56e, Obp56g, Obp56i* | *df/ore* | 6 | 15 | 29 | 0.00284 | 0.2 |
|  |  | *Bal/ore* | 17 | 17 | 16 |  |  |
| **27354** | *None* | *df/ore* | 15 | 22 | 13 | 0.07136 | 0.39 |
|  |  | *Bal/ore* | 24 | 18 | 8 |  |  |
| **26554** | *Obp57a, Obp57b, Obp57e* | *df/ore* | 4 | 8 | 13 | 0.0581 | 0.25 |
|  |  | *Bal/ore* | 9 | 9 | 7 |  |  |
| **7552** | *None* | *df/ore* | 11 | 33 | 6 | 0.3375 | 0.58 |
|  |  | *Bal/ore* | 16 | 27 | 5 |  |  |
| **7780** | *Obp22a* | *df/ore* | 7 | 21 | 22 | 0.005404 | 0.21 |
|  |  | *Bal/ore* | 17 | 22 | 11 |  |  |
| **7779** | *Obp22a* | *df/ore* | 10 | 16 | 24 | 5.22E-05 | 0.12 |
|  |  | *Bal/ore* | 26 | 17 | 7 |  |  |
| **7144** | *None* | *df/ore* | 24 | 10 | 16 | 0.09637 | 0.47 |
|  |  | *Bal/ore* | 29 | 12 | 9 |  |  |
| **24968** | *Obp83cd* | *df/ore* | 13 | 19 | 18 | 0.007646 | 0.25 |
|  |  | *Bal/ore* | 24 | 18 | 8 |  |  |
| **7778** | *None* | *df/ore* | 26 | 17 | 7 | 0.6518 | 1.06 |
|  |  | *Bal/ore* | 28 | 14 | 8 |  |  |
| **24346** | *Obp83cd* | *df/ore* | 12 | 9 | 19 | 0.01113 | 0.28 |
|  |  | *Bal/ore* | 25 | 14 | 11 |  |  |
| **24345** | *None* | *df/ore* | 18 | 20 | 12 | 0.3642 | 0.71 |
|  |  | *Bal/ore* | 19 | 22 | 9 |  |  |
| **7443** | *None* | *df/ore* | 23 | 18 | 9 | 0.1377 | 0.43 |
|  |  | *Bal/ore* | 30 | 15 | 5 |  |  |
| **7722** | *obp19b* | *df/ore* | 6 | 19 | 25 | 4.65E-05 | 0.1 |
|  |  | *Bal/ore* | 22 | 19 | 9 |  |  |
| **25738** | *None* | *df/ore* | 12 | 14 | 24 | 0.2636 | 0.65 |
|  |  | *Bal/ore* | 14 | 18 | 18 |  |  |

**Table S9.** Collection details for the eight surviving lines of *D. orena*.

| **Line** | **Collection site** | **Year** | **Collector** |
| --- | --- | --- | --- |
| Cameroon | Bafut N’Guemba, Cameroon | 1975 | J.R. David |
| Bioko1 | Balacha Norte, Bioko | 2013 | D.R. Matute |
| Bioko2 | Balacha Norte, Bioko | 2013 | D.R. Matute |
| Bioko3 | Balacha Norte, Bioko | 2013 | D.R. Matute |
| Bioko4 | Balacha Norte, Bioko | 2013 | D.R. Matute |
| Bioko5 | Balacha Norte, Bioko | 2013 | D.R. Matute |
| Bioko6 | Balacha Norte, Bioko | 2013 | D.R. Matute |
| Bioko7 | Lago Biao, Bioko | 2013 | D.R. Matute |
